# Exact Non-Markovian Quantum Dynamics on the NISQ Device Using Kraus Operators

**DOI:** 10.1021/acsomega.3c09720

**Published:** 2024-02-15

**Authors:** Avin Seneviratne, Peter L. Walters, Fei Wang

**Affiliations:** † Department of Physics and Astronomy, George Mason University, 4400 University Drive, Fairfax, Virginia 22030, United States; ‡ Department of Chemistry and Biochemistry, George Mason University, 4400 University Drive, Fairfax, Virginia 22030, United States; § Quantum Science and Engineering Center, George Mason University, 4400 University Drive, Fairfax, Virginia 22030, United States

## Abstract

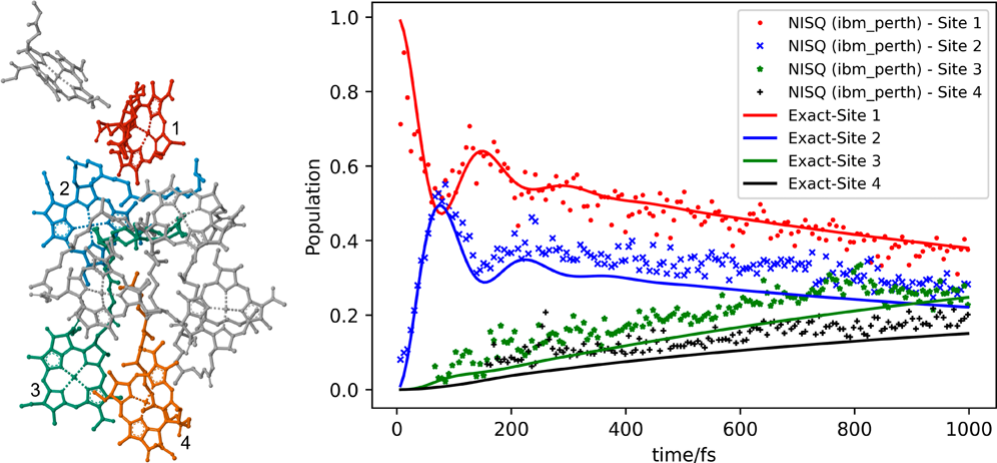

The theory of open quantum systems has many applications ranging from simulating quantum dynamics in condensed phases to better understanding quantum-enabled technologies. At the center of theoretical chemistry are the developments of methodologies and computational tools for simulating charge and excitation energy transfer in solutions, biomolecules, and molecular aggregates. As a variety of these processes display non-Markovian behavior, classical computer simulation can be challenging due to exponential scaling with existing methods. With quantum computers holding the promise of efficient quantum simulations, in this paper, we present a new quantum algorithm based on Kraus operators that capture the exact non-Markovian effect at a finite temperature. The implementation of the Kraus operators on the quantum machine uses a combination of singular value decomposition (SVD) and optimal Walsh operators that result in shallow circuits. We demonstrate the feasibility of the algorithm by simulating the spin-boson dynamics and the exciton transfer in the Fenna–Matthews–Olson (FMO) complex. The NISQ results show very good agreement with the exact ones.

## I Introduction

Open quantum system dynamics has gained increasing research interest due to its direct applications to quantum dynamics in the condensed phase,^1−3^ transport properties,^4,5^ quantum biology,^6−8^ and quantum error correction.^9^ Recent advances have uncovered many intriguing phenomena, such as environment-assisted exciton transfer,^10−14^ topological state preparation by reservoir engineering,^15,16^ information backflow,^17,18^ etc. Particularly for the non-Markovian bath, unique features exist, including coherence trapping,^19^ enhanced quantum entanglement,^20^ and the emergence of noncanonical equilibrium states.^21−23^ Accurate and efficient modeling of non-Markovian quantum dynamics has important ramifications in chemistry for studying charge transfer reactions in solutions^24^ and heterojunctions^25−27^ as well as exciton transfer in macromolecules and molecular aggregates.^28,29^ Due to the quantum mechanical nature of these processes, classical computer simulations can be prohibitively expensive due to the exponential scaling with the number of degrees of freedom and non-Markovianity.

Since the insightful suggestion by Feynman^30^ and the pioneering demonstration by Lloyd,^31^ numerous studies have emerged in seeking efficient quantum algorithms for quantum simulations.^32−38^ As for open quantum systems, although a wealth of literature exists for Markovian dynamics, including the construction of a universal set of semigroup generators^39,40^ and the development of various techniques for efficient Lindblad dynamics simulation,^41−51^ quantum algorithms for non-Markovian evolution are much less explored and are still in the nascent stage of advancement. Notable studies include Sweke et al.^52^ who considered locally indivisible maps that are capable of describing non-Markovian systems and Head-Marden et al.^53^ who used ensembles of Lindblad trajectories originating from different times to capture the non-Markovian behavior. Wang et al.^54^ constructed non-Markovian superoperators from the integrated generalized quantum master equation (GQME) and implemented them on NISQ devices through Sz.-Nagy dilation. Recently, a path integral based algorithm is proposed that encodes the Feynman–Vernon’s influence functional on a quantum computer.^103^

The focus of this paper is to present a new quantum algorithm that captures the exact non-Markovian dynamics based on the construction of Kraus operators. We explicitly resort to Feynman’s path integral formulation for generating the non-Markovian evolution. Adding to the existing pool of methods, our approach highlights two advantages. First, by implementing Kraus operators instead of superoperators on quantum machines, the propagation scheme is equivalent to time-evolving the wave function instead of the density matrix. As a consequence, it cuts the number of qubits needed by half. Second, by employing singular value decomposition (SVD), the nonunitary part is solely carried over to the diagonal matrix, which has efficient circuit implementation on quantum computers. These two merits lead to a shallow circuit structure that is amenable to simulating non-Markovian quantum dynamics using multiple qubits on NISQ devices. The organization of the paper is as follows. In Section II, we briefly review the operator-sum approach to open quantum system dynamics. In Section III, we describe the path integral techniques we use to construct exact non-Markovian superpropagators. In Section IV, we outline the procedures to construct Kraus operators from the superoperators. In Section V, we describe the unitarization of the Kraus operators using SVD and Walsh operators. In Section VI, we present simulation results on the NISQ device with two application cases, one based on the spin-boson model and the other based on the exciton transfer dynamics in the FMO complex. In Section VII, we offer concluding remarks.

## II Operator-Sum Approach for Open Quantum Systems

The time evolution of the density matrix is described by
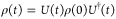
1where ρ refers to the total density matrix of the system and bath and *U*(*t*) is the time evolution operator. In many cases, we are only interested in the dynamics of a subsystem, and the reduced dynamics is achieved by tracing out the environment’s degrees of freedom. Without loss of generality, it is often assumed that the initial density matrix takes the tensor product form
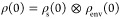
2Then, the reduced density matrix at time *t* is
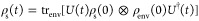
3ρ_env_(0) is expressed by its eigenstates
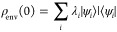
4Equation 3 becomes
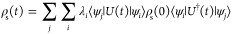
5

Define
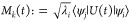
6called the Kraus operator,^55^ the reduced density matrix can be written as an operator-sum representation
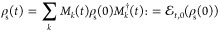
7which defines a linear map  that is complete positive and trace-preserving (CPTP). It can be proven that for a subsystem spanning the Hilbert space of dimension *d*, at most *d*^2^ numbers of Kraus operators are needed to completely mock up the CPTP map .^9^

The advantage of the Kraus operator representation is that the propagation scheme is analogous to the wave function propagation instead of the vectorized density matrix, therefore automatically reducing the number of qubits required by half and in turn shortening the circuit depth. Specifically, suppose ρ_s_(0) has the eigen decomposition ρ_s_(0) = ∑_*i*_σ_*i*_|φ_*i*_⟩⟨φ_*i*_|, then, the propagation can be achieved by each *M*_k_(*t*)|φ_*i*_⟩. Notwithstanding the advantage, one major concern of its quantum computing implementation is that *M*_k_(*t*) is not unitary, and therefore, additional steps are required to convert it to a unitary operation. Schemes for converting nonunitary operators to unitary ones exist,^45,47,49,56−58^ and in Section V, we will describe in detail the specific implementation used in this paper.

## Generation of the Linear Map  for Exact Non-Markovian Dynamics

III

We resort to the framework of a multistate system linearly coupled to its harmonic bath in the following discussion, for it has been shown to be widely applicable for simulating quantum dynamics in many condensed-phase systems.^3,24,25,28,59,60^ In Feynman’s path integral formulation,^61^ the time evolution of the reduced density matrix has the form
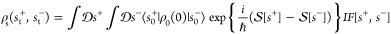
8where
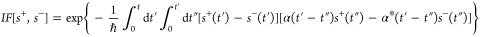
9is Feynman–Vernon’s influence functional,^62,63^ with the *s*^+^ and *s*^–^ being the forward and backward paths, respectively, *S*[s^+^] and *S*[s^–^] are the action integrals of the free system propagation, and ⟨*s*_0_^+^|ρ_0_(0)|*s*_0_^–^⟩ is the initial state. The nonlocal memory kernel α(*t*′ – *t*″) is the root of non-Markovianity and can be obtained from the bath response function
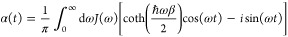
10where the spectral density *J*(ω) is defined as
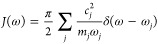
11Here, ω_*j*_ is the collective mode of the bath and *c*_*j*_ is the coupling strength between the system and bath.

Equation 8 defines an exact linear map  for non-Markovian quantum dynamics of the subsystem. Several numerically efficient implementations are available, such as QuAPI,^64,65^ QCPI,^66−68^ SMatPI,^69−72^ HEOM,^73−75^ TEMPO,^76−78^ TNPI,^79,80^ etc. In this work, we use TNPI^79^ to generate the linear map (i.e., the superoperator).

## IV From the Superoperators to the Kraus Operators

In this section, we outline the procedures for converting the superoperator to Kraus operators. By definition, a superoperator operates on a matrix, whereas an operator (or matrix) acts on a vector. It would be convenient to work in the matrix-vector scheme, and therefore, the initial step is to vectorize the density matrix, so that the superoperator can be expressed as a matrix. In this scheme, the superoperator acting on the density matrix can be written as
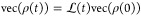
12where  is the matrix representation of the linear map . The conversion of the superoperator  to an operator-sum representation relies on the construction of the Choi matrix^81,82^ through the involution operation on , which is defined as
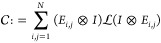
13in which *E*_*i,j*_ is a basis of an *N* × *N* matrix, with a “1” in the *i*, *j*th positions and zeros elsewhere. This Choi matrix is guaranteed to be positive and Hermitian for a CPTP map and therefore has an eigen decomposition with positive eigenvalues
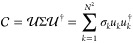
14

The vectorized Kraus operator *M*_*k*_ is related to this decomposition by
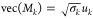
15Therefore, the matrix *M*_*k*_ can be reconstructed by taking the first *N* elements in ***u***_*k*_ to be the first column, the *N* + 1 to 2*N* elements in ***u***_*k*_ to be the second column, etc.

## V Unitarization of the Kraus Operators

Useful schemes have been proposed to convert nonunitary operations into unitary ones that can be implemented on quantum computers. One is based on the block encoding in which the nonunitary part is embedded in a larger unitary matrix. The implementation involves ancilla qubits, and the measurement related to the nonunitary operator is usually nondeterministic. Common block-encoding schemes include Stinespring dilation,^57^ Sz.-Nagy dilation,^58,83^ quantum signal processing (QSP),^56,84,85^ etc. Another popular scheme, called quantum imaginary time evolution (QITE),^49,50^ is based on a mapping between a nonunitary operator and a parametrized unitary operator, in which the parameter is solved by an algebraic linear equation. In this paper, we unitarize the Kraus operator *M_k_* through singular value decomposition (SVD). Specifically,
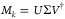
16where *U* and *V* are unitary matrices that can be directly implemented on a quantum computer. The ∑ matrix is a nonunitary diagonal and can be dilated to a unitary one in the following way.^47^ First, a unitary operator associated with the elements of ∑ is defined by
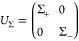
17where
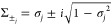
18with σ_*j*_ being the elements of ∑. It is worth mentioning that |σ_*j*_| is always smaller than 1, guaranteed by the fact that the CPTP map  is always contractive.^86^ As the dilation is needed only for the diagonal instead of a general matrix, the circuit depth is expected to be short. Its advantage is further illustrated in Tables 1 and 2.

**Table 1:** Complexity Comparison of the Walsh Operator Approach vs Direct Compilation of the Diagonal Matrix for the 2 qubit and 3 qubit Problems in Section VI

	2 qubits		3 qubits	
	Walsh operator	direct compilation	Walsh operator	direct compilation
circuit depth	4	6	13	100
number of CNOT	2	2	9	35

**Table 2:** Complexity Comparison of SVD vs Sz.-Nagy Dilation for the 2 qubit and 3 qubit Problems in Section VI

	2 qubits		3 qubits	
	SVD	Sz.-Nagy dilation	SVD	Sz.-Nagy dilation
circuit depth	11	14	46	123
number of CNOT	2	2	15	36

**Figure 1:**
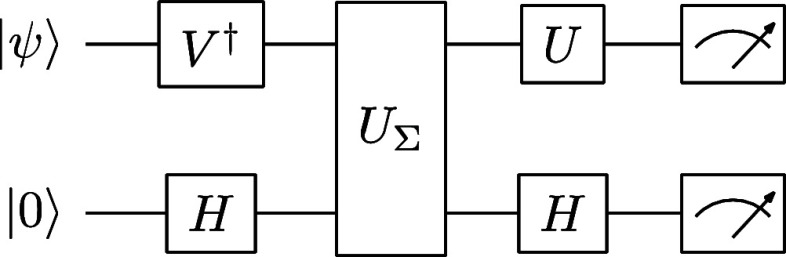
Circuit for SVD.

The circuit construction for the SVD^47^ is shown in Figure 1, where the ancilla qubit implements the Hadamard gates.

The resulting states are
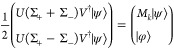
19in which the ancilla in the |0⟩ state implements *M*|ψ⟩, whereas the |1⟩ state measurement (labeled |φ⟩) is discarded. The measurement results show the statistics of *M*_*k*_|ψ⟩⟨ψ|*M*_*k*_^†^ in the computational basis, which is exactly the element in the operator-sum representation in eq 7.

The diagonal unitary, eq 18, can be implemented efficiently on a quantum computer using the Walsh series representation.^87^ Below, we summarize briefly the key construction. First, the integer *j*, *k* ∈ [0, 2^*n*–1^] is expressed in its binary and dyadic expansion, respectively
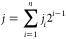
20
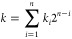
21where *n* is the number of qubits required to implement eq 17.

A matrix *W*_*jk*_ and a vector *f*_*k*_ are defined as
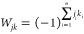
22
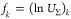
23

The Walsh coefficient *a*_*j*_ is obtained from the Walsh–Fourier transform of *f_k_*
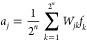
24

Then, the unitary diagonal *U*_∑_ can be expressed as
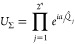
25with the Walsh operator *Q̂*_*j*_ given as the tensor product of Pauli Z gates
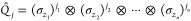
26

The circuit for the exponentiation of the tensor product of Pauli gates can be constructed^9^ and further optimized^87,88^ with the Gray code ordering to minimize the number of CNOT gates.

To validate the effectiveness of the Walsh series approach, we compare the gate complexity (circuit depth and the gate counts) using the optimal Walsh operators with that of the direct compilation of the unitary diagonal on Qiskit,^89^ all decomposed to the native gate sets of *X*, , *R*_Z_, and CNOT and complied to the topology of *ibm_perth* (shown in Figure S1). Table 1 gives the summary, and the circuits are shown in the Supporting Information (SI), Figures S2–S5.

We also compare the gate complexity of using SVD for unitarization of the Kraus operator to that of Sz.-Nagy dilation, compiled to the *ibm_perth* topology, and the results are shown in Table 2. The circuits are given in the SI, Figures S6–S9.

From a more quantitative perspective, for an *n* qubit problem, the number of single and CNOT gates scales as *O*(*n*^2^4^*n*^),^9^ which is the cost of Sz.-Nagy dilation. In the SVD approach, only the diagonal needs to be dilated, and the compilation of the two unitary matrices involves one qubit less and therefore only scales as *O*((*n* – 1)^2^4^(*n*−1)^). The dilated diagonal with the Walsh operator implementation scales at most *O*(2^*n*^).^87^ Therefore, it is no surprise that the SVD plus Walsh operator approach outperforms the Sz.-Nagy dilation.

## VI Results and Discussion

### VI.I Two-Level System

In this subsection, we present the simulation results for the spin-boson model,^62^ with the system-bath Hamiltonian given by
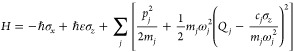
27

**Figure 2:**
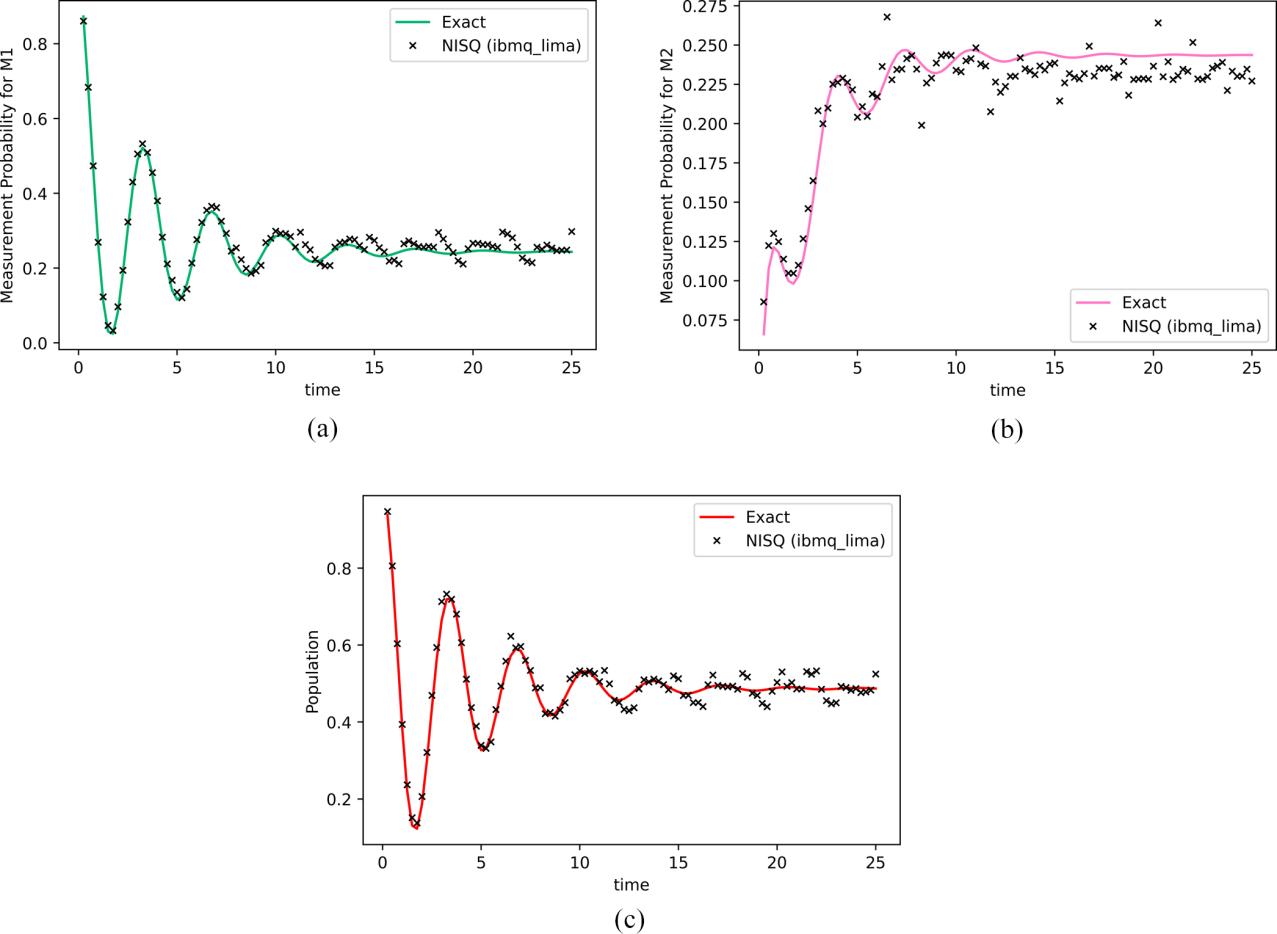
(a, b) Measurement results for the non-zero Kraus operators *M_k_* and (c) population dynamics with parameters Ω = 1, ϵ = 0, ξ = 0.1, ω_c_ = 7.5, and β = 5. (The time is in atomic unit.) The crosses are the results from *ibm_lima*, and the curves are the exact results. Each time point is obtained by 20,000 shots.

We choose the spectral density to have the Ohmic form
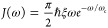
28which gives a continuous version of eq 11. It is worth mentioning that for a real molecular environment with discrete modes that resemble the ohmic form, the discretization of eq 28 gives a direct relationship between ξ, ω_c_ and *c*_*j*_, and ω_*j*_ in eq 27.^90,91^ In our simulation, the system bath parameters are with the following: tunneling frequency Ω = 1, asymmetry ϵ = 0, coupling strength ξ = 0.1, cutoff frequency ω_c_ = 7.5, and inverse temperature β = 5. In this parameter regime, we observe that out of the four Kraus operators, only two are significantly contributing. Figure 2a,b shows the measurement results for each of the two Kraus operators on the IBM NISQ device, and Figure 2c shows the population dynamics when adding them together. The NISQ results match well with the exact calculation from TNPI.^79^

### VI.II Exciton Dynamics for FMO

**Figure 3:**
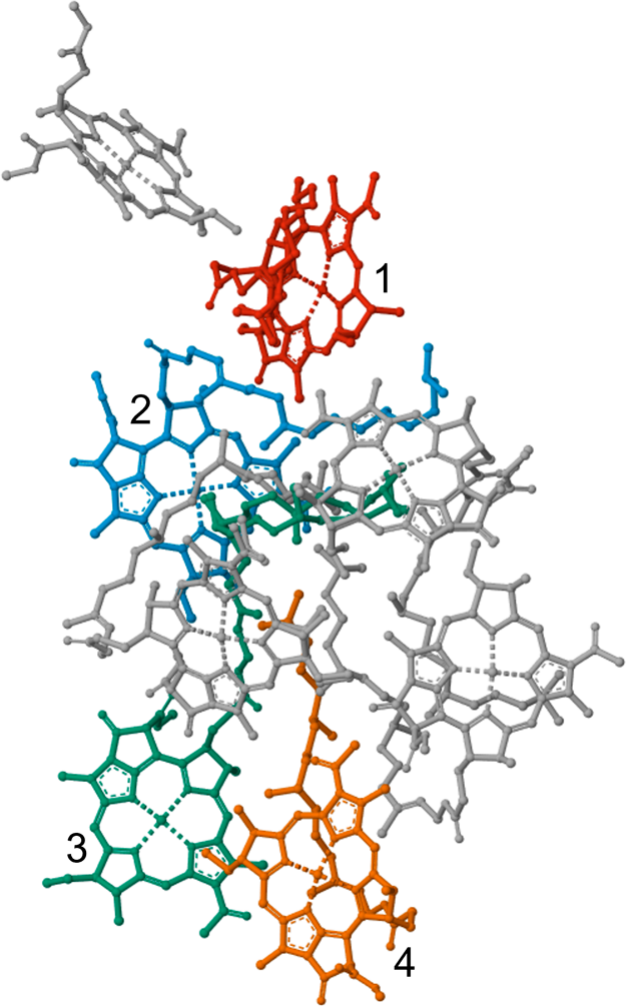
FMO complex and major pathway 1 → 2 → 3 → 4.

The FMO complex and its functional subsystems have received intense research investigation^92−97^ regarding its light harvesting efficiency and the quantum mechanical nature of the exciton transport. In this subsection, we study the exciton transfer dynamics in a major pathway 1 → 2 → 3 → 4 of the FMO, shown in Figure 3.

For this 4-site model, the Hamiltonian has the following Frenkel–Holstein^98−100^ form
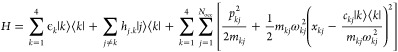
29

The system Hamiltonian is taken from the work done by Read et al.^101^ (see more details in SI eq (S.1))
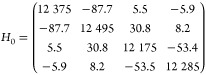
30

The values are in units of cm^–1^. Each exciton is coupled to its local harmonic bath that assumes the same Drude spectral density
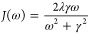
31with the reorganization energy λ = 35 cm^–1^ and phonon relaxation rate γ = 106.18 cm^–1^.^102^ The temperature of the simulation is 300 K.

**Figure 4:**
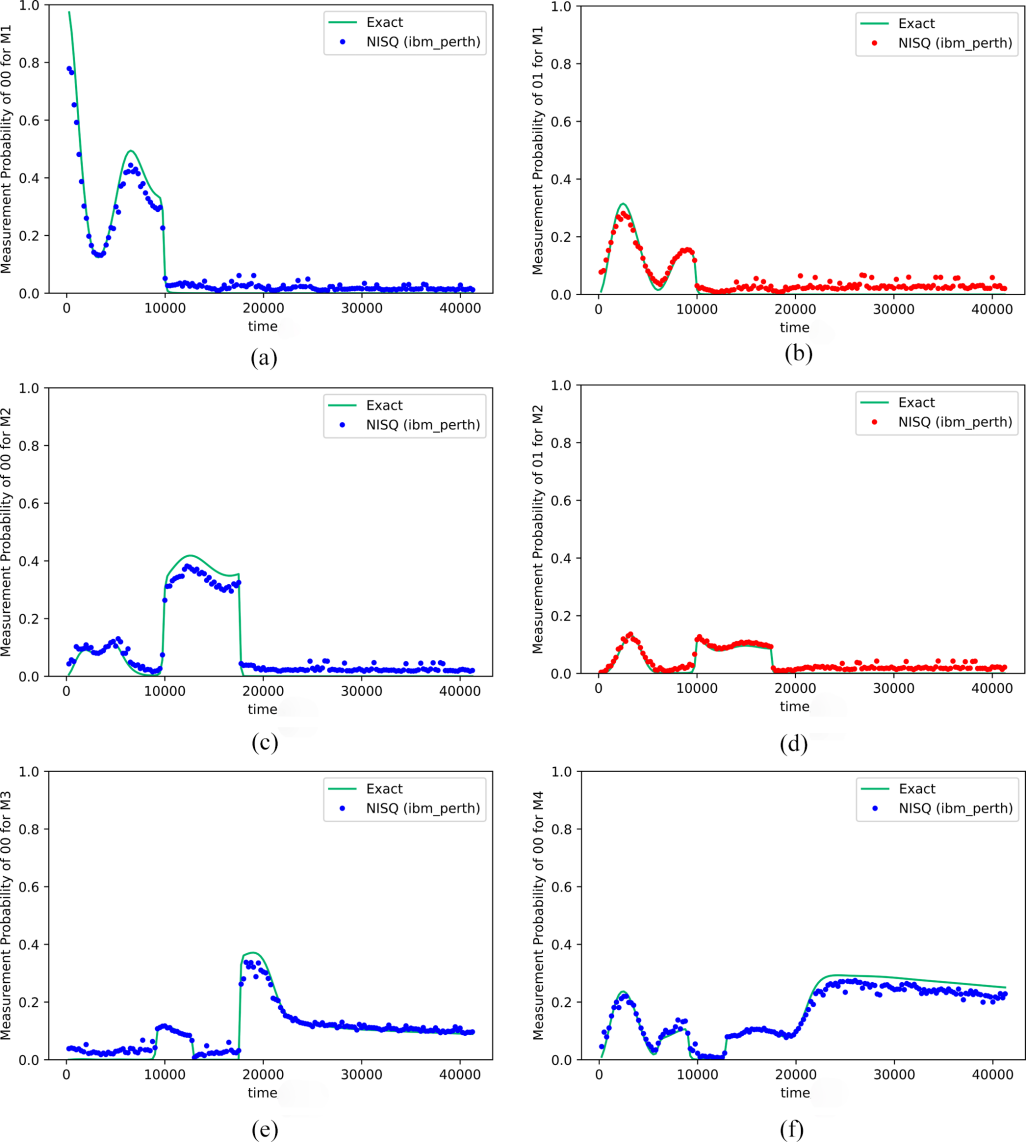
(a–f) Measurement results for the representative Kraus operators *M*_k_ for the FMO. The system Hamiltonian is taken from ref (83) and the bath parameters are λ = 35 cm^–1^ and γ = 106.18 cm^–1^ for the Drude spectral density^102^ and the temperature is 300 K. (The time is in atomic unit.) The dots are the results from *ibm_perth*, and the curves are the exact results. Each time point is obtained by 50,000 shots.

**Figure 5:**
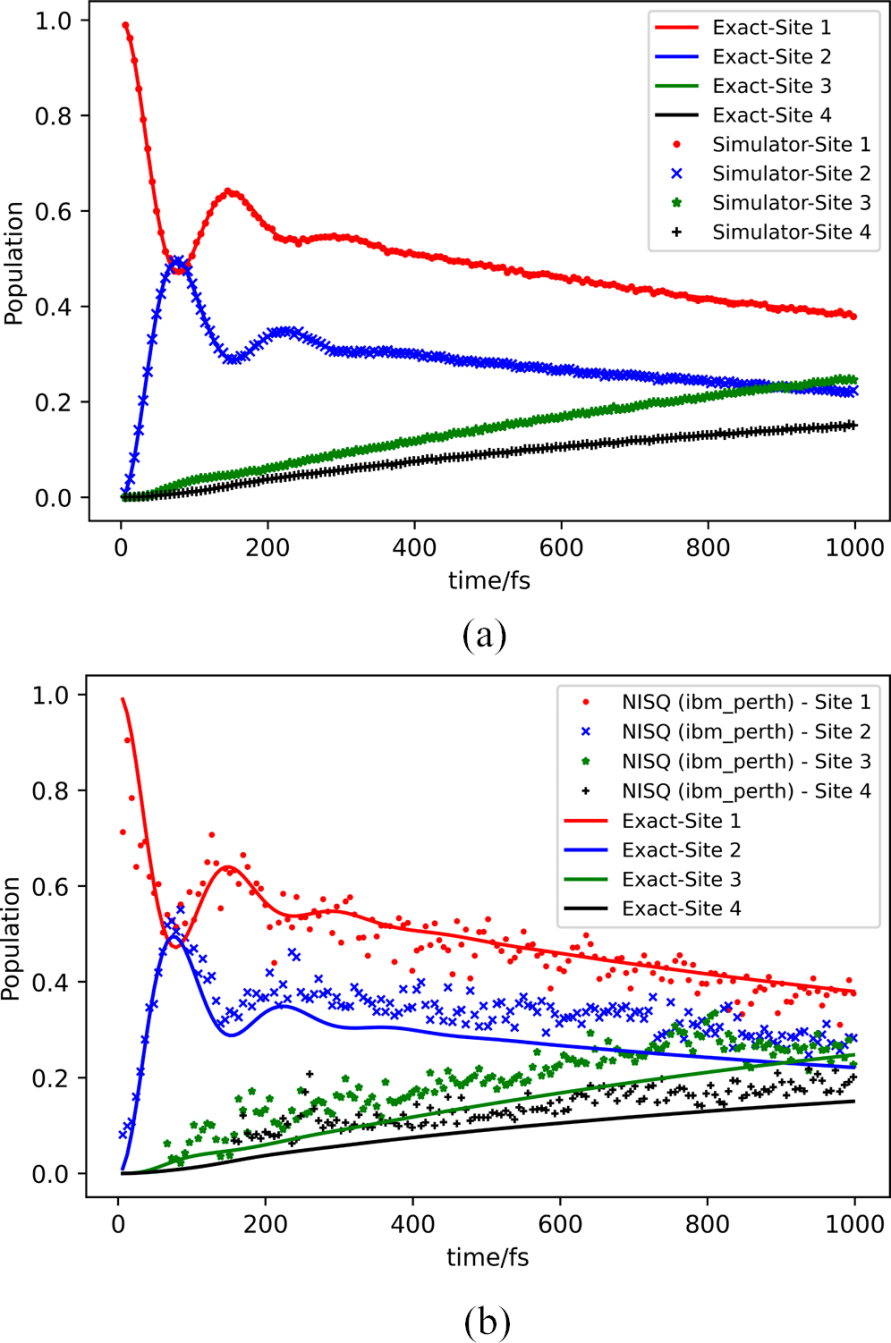
Population dynamics for a major pathway 1 → 2 → 3 → 4 in FMO on (a) simulators and (b) IBM device, with crosses representing the results from *ibmq_perth*. (The time unit is given in fs).

Among all 16 Kraus operators, only a handful are significantly contributing. Figure 4a–f shows some representative Kraus operator measurement results on the IBM NISQ device. With two qubits (plus one ancilla) representing the four sites, each Kraus operator has four measurement probabilities, labeled 00, 01, 10, and 00 in the Qiskit convention. It is not entirely clear to us why the Kraus operators have jumps at specific times. However, these discontinuous jumps cancel each other and produce smooth dynamics when adding them together. Figure 5a,b shows the population dynamics of the four sites by adding all the individual Kraus operators, with (a) being the simulator results and (b) being the real device. The NISQ results show quantitative agreement with the exact solution.

## VII Conclusions

We have demonstrated a feasible quantum algorithm for simulating exact non-Markovian dynamics on the NISQ device. The shallow circuit is achieved by three strategies. First, instead of implementing the superoperator directly, we choose to implement its associated Kraus operators. This is equivalent to propagating the wave function instead of the vectorized density matrix. Reducing the dimensionality from *n*^2^ to *n*, where *n* is the size of the wavevector, it automatically saves half of the qubits and as a consequence shrinks the circuit depth. Second, we employ SVD to encode the nonunitary Kraus operators. With two unitary matrices naturally coming out of this procedure, the dilation is only applied to the diagonal part. Third, we employ the optimal Walsh operators to implement the unitary diagonal. The combination of the SVD and the Walsh operator approach results in significant improvement in compilation complexity compared to the Sz.-Nagy dilation. As a general figure of merit for the above numerically exact algorithm, the number of non-zero Kraus operators often does not grow exponentially with the system size, therefore circumventing the costly measurement overhead. With respect to the spectral density, this theoretical framework allows an arbitrary form. In conclusion, the successful implementation on the NISQ device with the 4-site model suggests that the algorithm can potentially handle even larger Hilbert space (more qubits) without incurring too much noise.

## Supporting Information Available

Topology of *ibm_perth*, compiled circuits on *ibm_perth* for the diagonal matrix as well as the Kraus operators for the two-level and the four-site model, using both the singular value decomposition and direct compilation methods, and four-site system Hamiltonian values explained (PDF)
