# Supplementary material for: Exact Non-Markovian Quantum Dynamics on the NISQ Device Using Kraus Operators
Source: ACS Omega. 2024 Feb 15;9(8):9666–75. doi: 10.1021/acsomega.3c09720 (PMC10906042; doi:10.1021/acsomega.3c09720)
Supplement: Supplementary file 1 — ao3c09720_si_001.pdf [file ao3c09720_si_001.pdf]

Supporting Information

Exact non-Markovian quantum dynamics on NISQ device using Kraus operators

Avin Seneviratne[1], Peter L. Walters[2], Fei Wang[2,3,*]
[1]Department of Physics and Astronomy, George Mason University, Fairfax, Virginia 22030, USA
[2]Department of Chemistry and Biochemistry, George Mason University, Fairfax, Virginia 22030, USA
[3]Quantum Science and Engineering Center, George Mason University, Fairfax, Virginia 22030, USA

1. Circuits for the diagonal matrix and the Kraus operators.

All circuits are compiled to native gates X, sqrt(X), R_z, and CNOT, and conformed to the topology of *ibm_perth.*

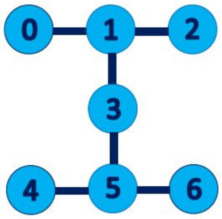

Figure S1. Topology of *ibm_perth*.

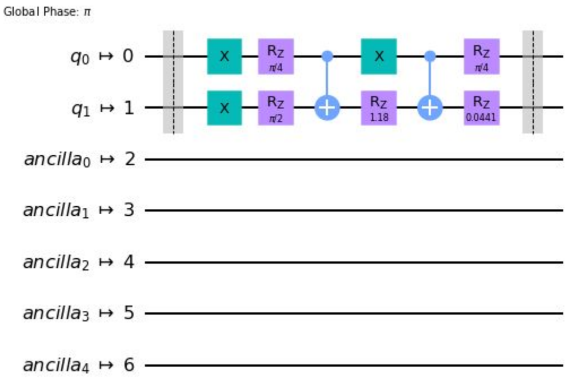

Figure S2. Circuit for the 4 by 4 diagonal, using direct compilation. (depth=6)

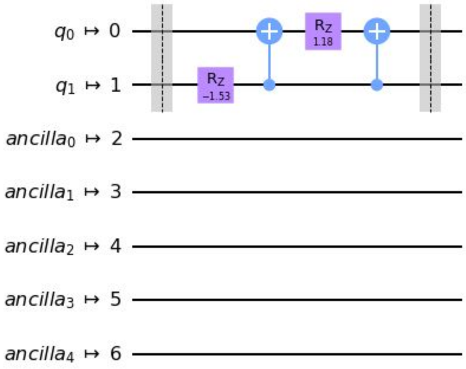

Figure S3. Circuit for the 4 by 4 diagonal, using Walsh operators. (depth=4)

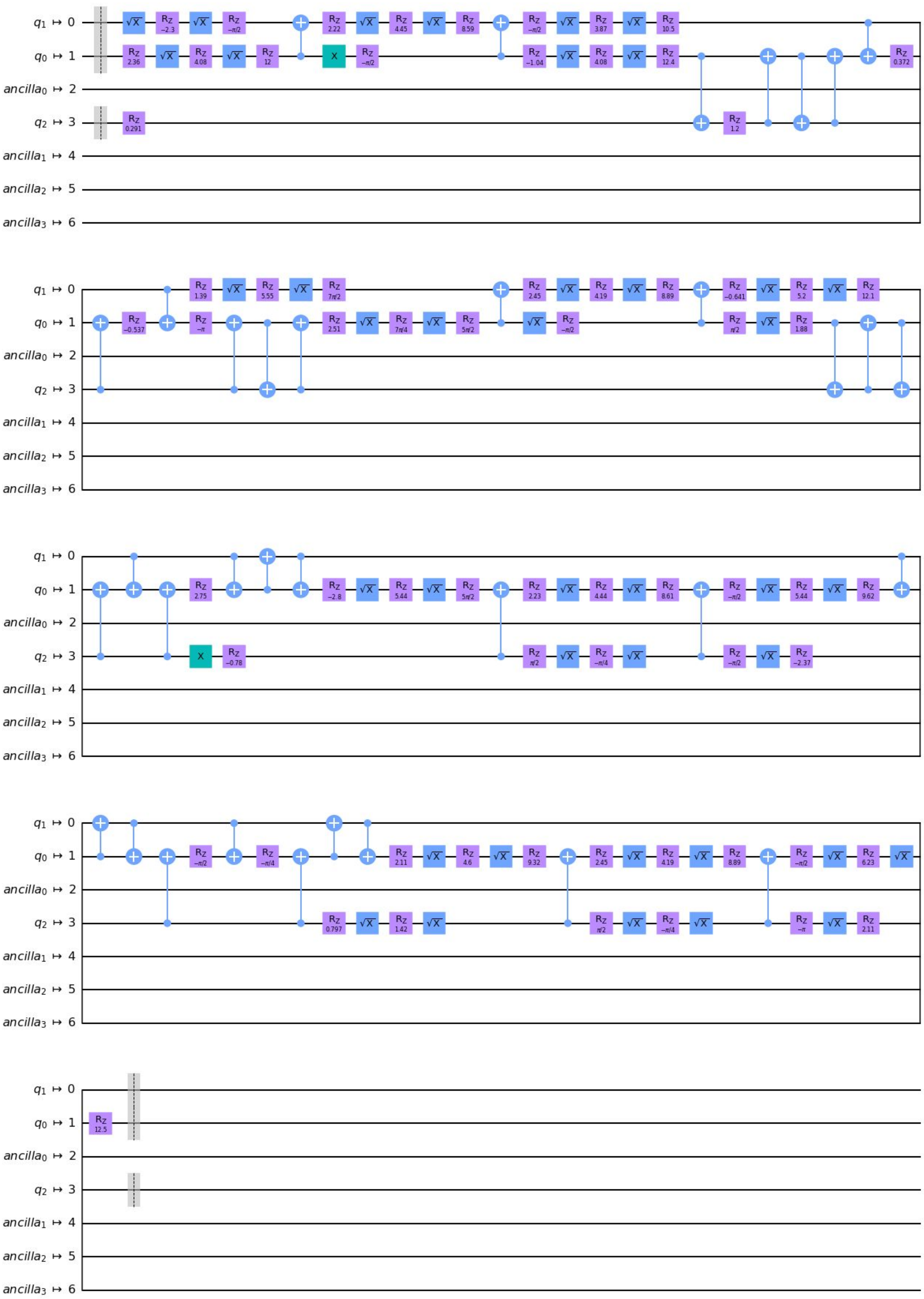

Figure S4. Circuit for the 8 by 8 diagonal, using direct compilation. (depth=100)

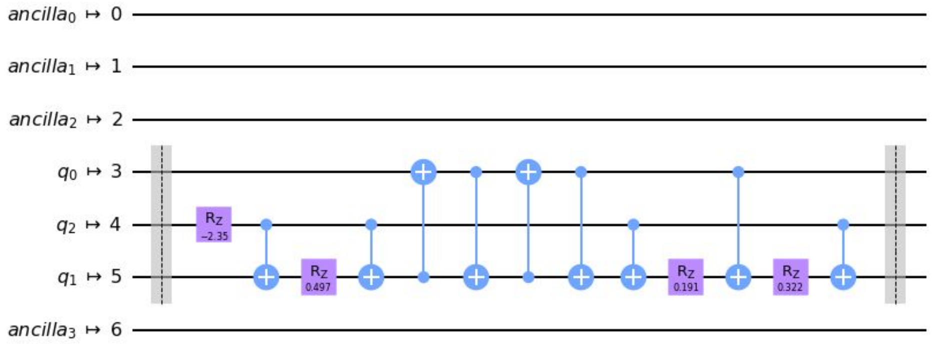

Figure S5. Circuit for the 8 by 8 diagonal, using Walsh operators. (depth=13)

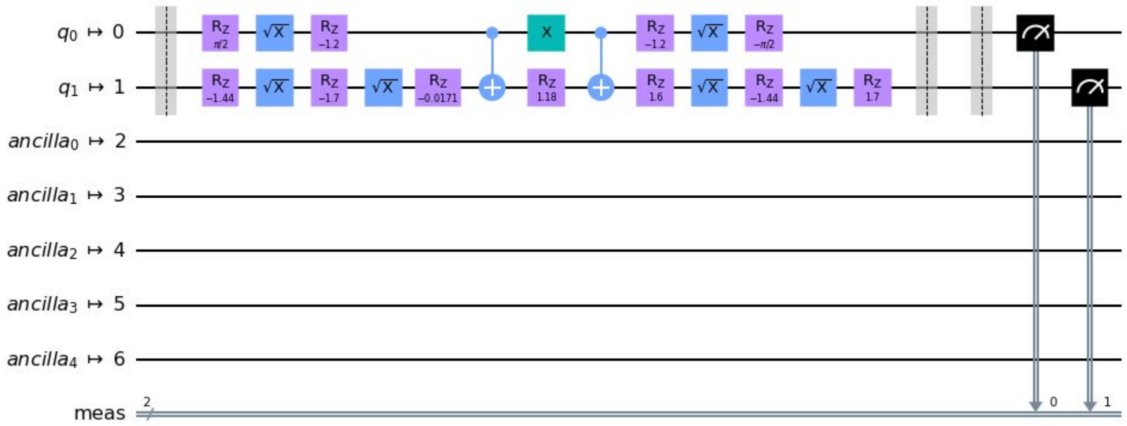

Figure S6. Circuit for the two-level system Kraus operator, using Sz.-Nagy dilation. (depth=14)

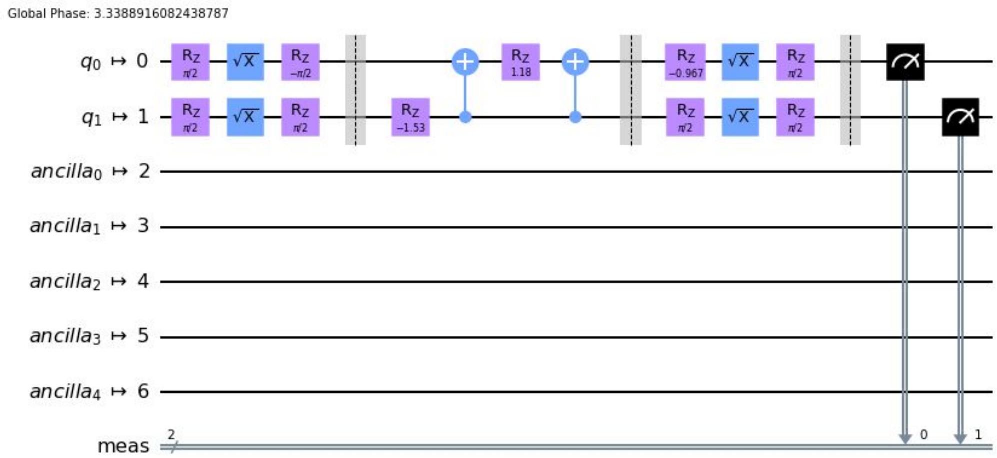

Figure S7. Circuit for the two-level system Kraus operator, using SVD. (depth=11)

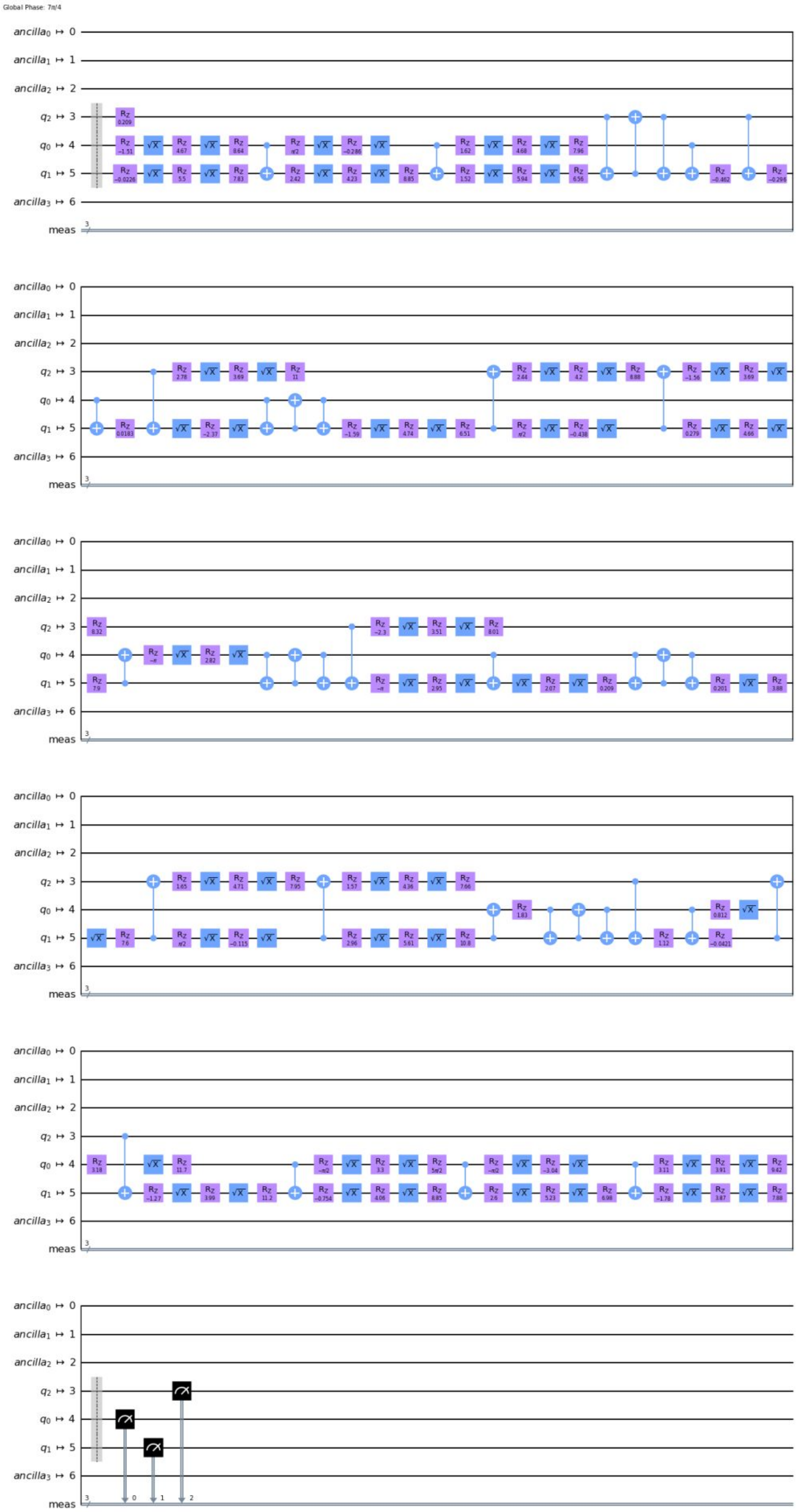

Figure S8. Circuit for the four-site model Kraus operator, using Sz.-Nagy dilation. (depth=123)

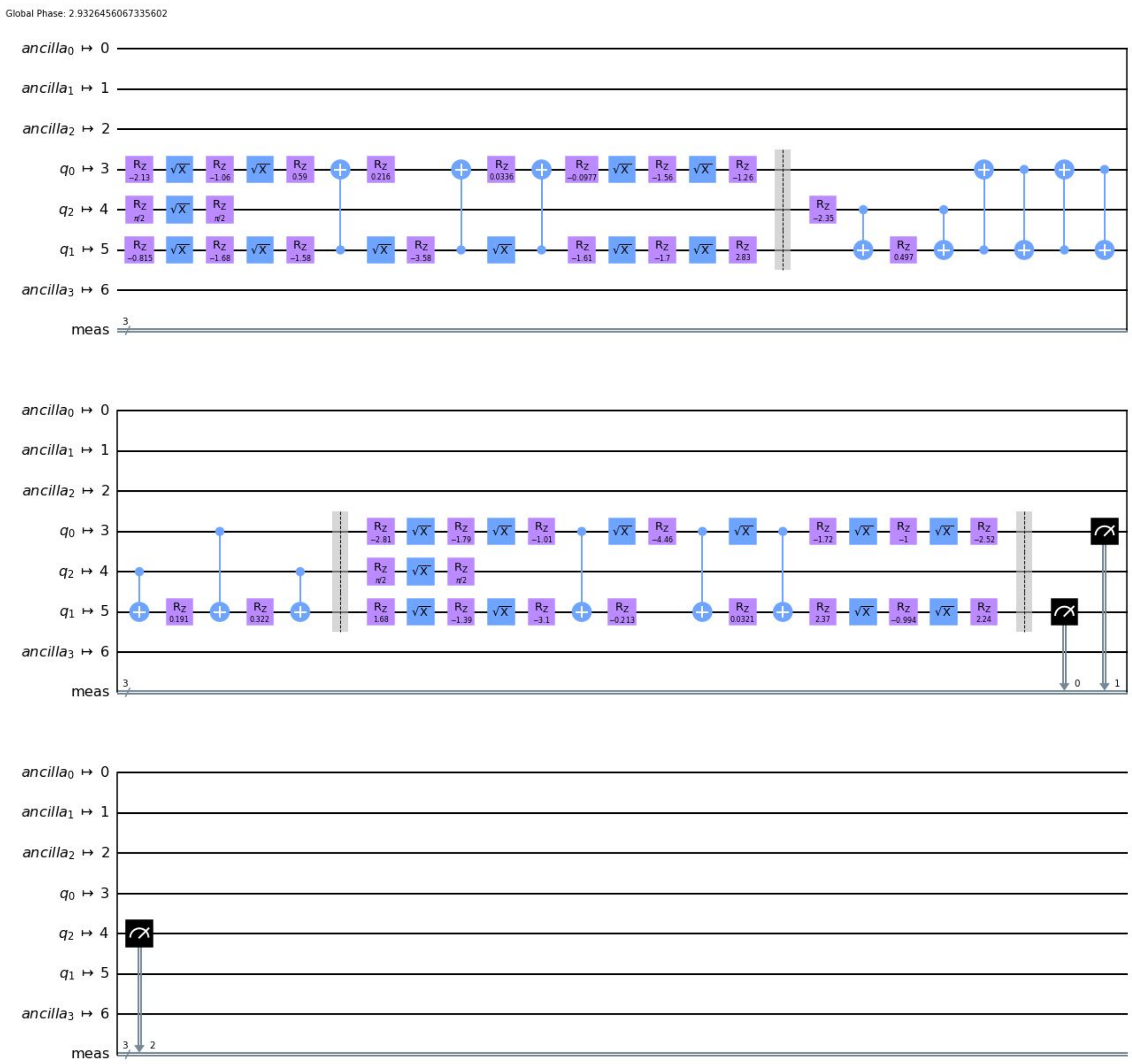

Figure S9. Circuit for the four-site model Kraus operator, using SVD. (depth=46)

2. System Hamiltonian for the 4-site model

The four-site system Hamiltonian[1] is obtained based on the values provided by Ref. 1. The site energies have been shifted by the bath's reorganization energy to account for QuAPI,[2] and therefore gives rise to the following:

$$H_0 = \begin{pmatrix} 12375 & -87.7 & 5.5 & -5.9 \\ -87.7 & 12495 & 30.8 & 8.2 \\ 5.5 & 30.8 & 12175 & -53.4 \\ -5.9 & 8.2 & -53.5 & 12285 \end{pmatrix} \#(\mathrm{S.1})$$

Reference

(1) Read, E. L.; Schlau-Cohen, G. S.; Engel, G. S.; Wen, J.; Blankenship, R. E.; Fleming, G. R. Visualization of Excitonic Structure in the Fenna-Matthews-Olson Photosynthetic Complex by

Polarization-Dependent Two-Dimensional Electronic Spectroscopy. *Biophys. J.* **2008**, *95* (2), 847–856. https://doi.org/10.1529/biophysj.107.128199.

(2) Topaler, M.; Makri, N. System-Specific Discrete Variable Representations for Path Integral Calculations with Quasi-Adiabatic Propagators. *Chem. Phys. Lett.* **1993**, *210* (4–6), 448–457. https://doi.org/10.1016/0009-2614(93)87052-5.
